# Broiler breeder putative lipid biomarkers associated with sperm mobility

**DOI:** 10.3389/fphys.2024.1504557

**Published:** 2024-11-15

**Authors:** A. Bond, K. M. Mills, C. R. Ferreira, I. Harford, B. Flack, J. A. Long, K. Diehl

**Affiliations:** ^1^ Department of Animal and Avian Sciences, University of Maryland, College Park, MD, United States; ^2^ Agricultural Research Service, Beltsville Agricultural Research Center, United States Department of Agriculture, Beltsville, MD, United States; ^3^ Bindley Bioscience Center, Purdue University, West Lafayette, IN, United States; ^4^ Cobb-Vantress Inc., Siloam Springs, AR, United States

**Keywords:** broiler breeder, semen, sperm mobility, lipidome, age

## Abstract

**Introduction:**

Biomarkers indicative of sperm mobility in broiler breeders would provide the ability to screen for fertility potential, with a positive correlation established between sperm mobility and fertilization potential. This study characterized the lipidome of seminal plasma (SP), sperm cell (SC), and whole semen (WS) isolated from broiler breeder roosters with different sperm mobility phenotypes across key timepoints of the semen production cycle.

**Methods:**

WS samples were collected from five high mobility roosters and five low mobility roosters during early, mid, and late semen production, with SP separated from SC by centrifugation. Using multiple reaction monitoring (MRM) profiling, a total of 3241 lipid species were identified in rooster semen across ten lipid classes. Metaboanalyst 6.0 was used to analyze the relative ion intensity for each lipid species due to sperm mobility phenotype through a t-test and due to timepoint through a one-way ANOVA, with lipid ontology enrichment analysis performed using LION. Metaboanalyst 6.0 was also used to perform biomarker analysis for the sperm mobility phenotype in WS samples.

**Results:**

Lipid class total abundance differed with sample type, sperm mobility phenotype, and timepoint. A total of 31, 99, and 112 lipid species were found to be different between low and high mobility males across timepoints in the SP, SC, and WS samples, respectively. Lipid ontology enrichment analysis revealed stark contrasts in lipid-based functions key to sperm survival, storage, and productivity between low and high sperm mobility phenotypes. Through biomarker analysis, 8 lipid species were identified as excellent sperm mobility biomarkers that could be detected in early and mid-semen production.

**Discussion:**

Timepoint based changes in lipid species were unique to each sperm mobility phenotype, with low sperm mobility roosters exhibiting a larger number of lipid species changes over the semen production cycle in the SP and SC when compared to high sperm mobility roosters. This is the first study to characterize poultry semen lipidome using MRM profiling. The lipid species identified between low and high sperm mobility roosters could be utilized in the poultry industry as potential biomarkers of fertility potential, with the ability to screen for the economical trait of fertility potential early in semen production.

## 1 Introduction

Semen biomarkers indicative of sperm mobility in broiler breeders would provide the ability to screen for fertility potential, with a positive correlation previously established between mobility and fertilization potential. Poor broiler breeder semen quality has many negative impacts on the poultry industry, including reduced fertility rates and increased housing costs to accommodate the number of roosters required to meet fertility requirements (Xue et al., 2022). Traditionally, poultry semen quality can be assessed through semen volume, sperm concentration, sperm viability, and subjective motility assessment, but these methods cannot be directly correlated to fertility potential (Donoghue et al., 1995). Methods that quantify poultry sperm function, such as sperm mobility assessment, and that provide potential biomarkers of fertility potential that can be utilized in the poultry industry, such as omic-based assessment, can improve overall flock male fertility at a faster rate than traditional methodology. The increase in the demand for broiler production has enhanced the need for efficient flock fertility rates. In a commercial setting, a single male will breed multiple females, making the impact of the rooster contribution on fertility rates greater than that of the hen.

The sperm mobility assay is an *in vitro* test that requires sperm to swim in a directional manner against resistance, which parallels the requirements *in vivo* for sperm to swim to sperm storage tubule (SST) temporary storage as well as from the SSTs to the ovum for fertilization. Sperm mobility is currently the only semen quality parameter directly associated with fertility potential in poultry. Further, roosters with low or high sperm mobility stay consistent in flock rankings through early and mid-timepoints of the semen production cycle. While the sperm mobility assay is informative, it is labor intensive and requires specialized equipment, which is not feasible in an industry setting. For seamless industry adoption, automated mid-high throughput methods are necessary to detect sperm mobility phenotypes, ideally during early semen production. Lipidomics is an emerging omic-based technology that could allow the poultry industry to economically screen for sperm mobility phenotypes if reliable biomarkers could be identified. Lipids were selected as the basis for this research as sperm cell and seminal plasma lipids are integral to proper sperm function, including processes involving viability, SST storage, and ovum fertilization.

Sperm cell lipids contribute to membrane composition, signaling, metabolism, and a myriad of other functions that aid in sperm mobility. These include sperm cell membrane integrity and fluidity, lipid based signaling mechanisms, and energy sources that can be utilized through lipid metabolism mechanisms. Lipids are available in poultry sperm in the form of polyunsaturated fatty acids (PUFAs), playing key roles in sperm membrane integrity and function. In particular, arachidonic acid, docosatetraenoic acid, and the other members of the n-6 family contribute to membrane sperm fluidity and flexibility for sperm functions during *in vitro* storage (Cerolini et al., 2003). Also, docosahexaenoic acid and its respective n-3 family facilitate sperm development, motility, and viability (Khoshvaght et al., 2016). Sperm plasma membrane lipids also impact sperm cryotolerance during cryopreservation procedures (Musa et al., 2021). Phospholipid class abundances in spermatozoa throughout the reproductive period of male chickens has been previously correlated with *in vivo* fertility rates*,* with phosphatidylserine and phosphatidylcholine displaying a pattern of changes with age positively and negatively, respectively, in relation to the changes of fertility (Cerolini et al., 1997). Shifts in sperm cell lipid composition impact sperm membrane fluidity and diffusion, directly influencing sperm nutrient uptake from the seminal plasma and/or oviductal fluids.

The biochemical characteristics and physiological roles of the various seminal plasma components in birds (carbohydrates, lipids, amino acids, hormones, and proteins) are poorly understood (Blesbois et al., 2004). The total lipid content of seminal plasma is lower than the total lipid content seen in sperm cells, with a lower proportion of phosphatidylcholine and high levels of cholesterol esters and triglycerides (Longo, 2014; Zaniboni and Cerolini, 2009; Douard et al., 2000). Though lipid functions in the seminal plasma have not been fully elucidated, poultry seminal plasma lipid content has been previously associated with fertility rates and has been shown to change with age. Shifts in seminal plasma lipid composition through dietary intervention has been utilized to increase semen fertilizing ability (Blesbois et al., 1997; Blesbois et al., 2004). However, seminal plasma lipid concentrations are impacted by age, with concentrations higher in seminal plasma of older roosters than in younger roosters (Kelso et al., 1996). Additionally, the presence of seminal plasma greatly impacts the success of bird sperm fertilizing capacity during *in vitro* semen biotechnologies, indicating potential impacts on sperm mobility (Blesbois et al., 2004).

Early semen screening for sperm mobility would allow the poultry industry to minimize costs associated with males exhibiting low fertility potential. The focus of this study is to characterize the lipidome of seminal plasma (SP), sperm cells (SC), and whole semen (WS) in broiler breeders with different sperm mobility phenotypes. In addition, this study assessed how the lipidome changes across the male production cycle in these sample types for low and high mobility phenotypes. This is the first study to characterize the lipidome of poultry semen fractions using multiple reaction monitoring (MRM) profiling. Through this analysis, potential lipid biomarkers of sperm mobility will also be identified, which could be utilized in the broiler breeder industry for selecting rooster with improved sperm mobility and, consequently, improved fertility potential.

## 2 Materials and methods

All procedures in this study were approved by the Institutional Animal Care and Use Committee of the Beltsville Animal Research Center (BARC), United States Department of Agriculture. Forty Cobb broiler breeder roosters were obtained at 22 weeks of age and used for this study. Roosters were housed individually at BARC from 22 to 55 weeks of age. Throughout the study duration, roosters had *ad libitum* access to water and were fed based on Cobb broiler breeder management guidelines. At 22 weeks of age, rooster photoperiod was increased to 10L:14D with weekly increases of 1 h of light until 16 h of light was reached to encourage the initiation of sexual maturation, inducing semen production. Semen was collected twice weekly from each rooster from 25 to 55 weeks of age, using the abdominal massage method to collect semen (Burrows and Quinn, 1935).

At 27–29 weeks of age, semen was collected and examined using the sperm mobility assay (Manier et al., 2019). Briefly, 10 μL of the collected semen sample is diluted with 3% sodium nitrate and a single wavelength photometer IMV Micro-Reader is used to measure absorbance to determine concentration. Mobility buffer is used to dilute the semen to 1 × 10^9^ sperm/mL prior to overlay of 300 μL of the diluted semen sample on a 6% Accudenz solution preheated to 41°C. Finally, absorbance is measured following a 5-minute cuvette incubation at 41°C to obtain a sperm mobility score. A total 4 sperm mobility screenings were performed during this period and the scores obtained for each screening was averaged for individual roosters. Roosters were classified as having low, average, and high sperm mobility, with the top 5 roosters used as the high mobility samples for this study and the bottom 5 roosters used as the low mobility samples for this study. Low, average, and high sperm mobility cutoffs were previously established. Low sperm mobility roosters used in this study had an average sperm mobility score of 0.256, while high sperm mobility roosters used in this study had an average sperm mobility score of 0.659.

Semen samples utilized in this study were collected from low and high sperm mobility roosters at 30, 42, and 55 weeks of age, corresponding to early, mid, and late timepoints during the semen production cycle. At each collection timepoint, a subset of the collected semen sample was snap frozen in liquid nitrogen and utilized as the WS samples described in this study. The remaining semen was separated by centrifugation to produce SC and SP samples described in this study. Briefly, semen samples were centrifuged at 2000 × g for 5 min at 4°C. The SP was removed as the supernatant and placed into a clean microcentrifuge tube, leaving behind the SC sample to be snap frozen in liquid nitrogen. The SP sample was centrifuged at 12,000 × g for 20 min at 4°C twice to remove remaining SC, prior to being snap frozen in liquid nitrogen. All samples were stored at −80°C until analysis.

Lipidomic analysis was performed using methods previously described (Reis et al., 2023). Briefly, lipids were extracted from samples using the Bligh and Dyer method (Bligh and Dyer, 1959), involving a methanol and chloroform extraction leading to sample separation into three phases consisting of the polar, protein, and organic (lipid) phase. The lipid phase was isolated and dried in a vacuum concentrator for 8 h, with dried pellets resuspended in 200 µL of acetonitrile, methanol, and ammonium acetate 3:6.65:0.35 (v/v/v) prior to a further 10X dilution of sample in solvent before direct injection. Multiple reaction monitoring (MRM) profiling was performed using a discovery phase followed by a screening phase. During the discovery phase, pooled samples were screened for the chemical classes of acylcarnitine (AC), cholesteryl ester (CE), ceramide (CER), phosphatidylcholine (PC), phosphatidylethanolamine (PE), phosphatidylinositol (PI), phosphatidylglycerol (PG), phosphatidylserine (PS), diacylglycerol (DG), and triacylglycerol (TG) lipids. DG and TG lipids were profiled using one fatty acyl neutral loss as a product ion for the MRM scan. The specific fatty acyl chain will be indicated next to the name of the class. For example, DG 16:0 means DG with a product ion compatible with palmitic acid. For the discovery phase, samples were pooled by sperm mobility phenotype, dried by nitrogen flow for 8 h prior, diluted in 200 µL of acetonitrile/methanol/ammonium acetate 300 mM 3:6.65:0.35 (v/v/v). Pooled samples were injected into the microautosampler (G1377A) in a QQQ6410 triple quadrupole mass spectrometer (Agilent Technologies, San Jose, CA) equipped with an ESI ion source. Initial chemical class data was processed using MSConvert 20 to convert profiling method data into mzML format. Unidentified MRM ion pairs were assigned tentative lipid class attributions based on associated functional group and biological information using Lipid Maps (http://www.lipidmaps.org/). A one-way ANOVA was used to compare lipid class total abundance across semen fractions using data generated through the discovery phase. Ions with values >30% in at least one of the samples compared to a blank within each profiling method were selected to be further analyzed in the screening phase.

Due to the large number of MRMs selected to be screened, individual samples were interrogated using three lists of MRMs, referred to as methods: M1 (CER, PC, and PE lipid species), M2 (TG lipid species), and M3 (AC, DG, CE, PS, PI, and PG lipid species). For screening phase data analysis, the relative ion intensity of a given MRM in each sample was calculated and analyzed by individual method. A two-way ANOVA was used to compare lipid class total abundance due to sperm mobility phenotype and timepoint using data generated through the screening phase. For both analyses, means were separated using the LSMeans statement in SAS. Relative intensity of MRM ion pairs was analyzed using MetaboAnalyst 6.0, with autoscaling data normalization. Student’s t-test analysis was used to identify MRMs that distinguish between low sperm mobility and high sperm mobility phenotypes, using an alpha of 0.05 of nominal *P*-value to identify differentially distributed lipids. Differentiating lipids were also subjected to biomarker analysis using classical univariate receiver operating characteristic (ROC) curve analysis with area-under-the-curve (AUC) value representative of biomarker potential. The following AUC scale was used to evaluate lipids as potential biomarkers: greater than or equal to 0.9 scored as excellent; greater than or equal to 0.8 but less than 0.9 scored as good; greater than or equal to 0.7 but less than 0.8 scored as fair; greater than or equal to 0.6 but less than 0.7 scored as poor; and less than 0.6 scored as fail (Xia et al., 2013). MRMs were also compared across timepoints by one-way ANOVA followed by Fisher *post hoc* analysis using Metaboanalyst 6.0. Differentiating lipid species identified between low and high sperm mobility samples as well as across timepoints within a given sperm mobility phenotype were subjected to functional annotation analysis using the target list mode of Lipid Ontology Enrichment Analysis (LION, http://lipidontology.com) (Molenaar et al., 2019; Molenaar et al., 2023). All lipid species detected within the appropriate fraction were inputted as the lipid background list for analysis.

## 3 Results

### 3.1 Lipid class distribution in semen fractions

**FIGURE 1:**
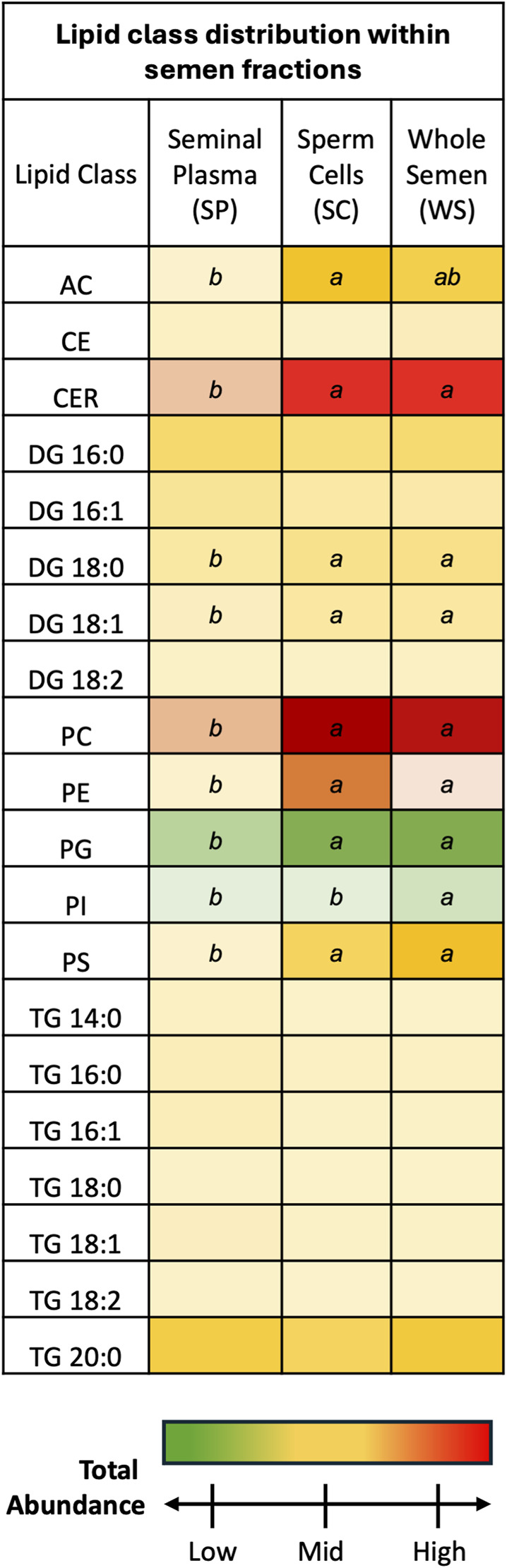
Discovery phase total abundance of lipids from 20 lipid classes. Ceramides and phosphatidyl cholines were most prevalent. Phosphatidyl glycerols and phosphatidylinositols were least prevalent. Fraction comparisons with statistically significant differences are indicated by different letters: ab *p* < 0.05. The following lipid classes were analyzed during the discovery phase: acylcarnitine (AC), cholesteryl ester (CE), ceramides (CER), diacylglycerol containing a 16:0 fatty acyl chain (DG 16:0), 16:1 (DG 16:1), 18:0 (DG 18:0), 18:1 (DG 18:1), and 18:2 (DG 18:2), phosphatidylcholine (PC), phosphatidylethanolamine (PE), phosphatidylglycerol (PG), phosphatidylinositol (PI), phosphatidylserine (PS), and triacylglycerol containing a 14:0 fatty acyl chain (TG 14:0), 16:0 (TG 16:0), 16:1 (TG 16:1), 18:0 (TG 18:0), 18:1 (TG 18:1), 18:2 (TG 18:2), and 20:0 (TG 20:0).

SP and SC samples exhibited unique lipid class total abundance profiles, with WS samples displaying aspects of both fractions (Figure 1). Overall, CER and PC lipid classes were most prevalent while PG and PI lipid classes were least prevalent. One-way ANOVA results examining lipid class total abundance differences due to fraction revealed significant differences in 8 out of the 10 lipid classes. The lipid classes that displayed significant differences are as follows: AC, CER, DG containing a 18:0 fatty acyl chain (DG 18:0) DG 18:0, DG 18:1, PC, PE, PG, PI, and PS. Total abundance differences due to fraction were not observed in a subset of the DG lipid classes as well as the CE and TG lipid classes. Post-hoc comparisons between SP and WS as well as SC and SP were significant for AC, CER, DG 18:0, DG 18:1, PC, PE, PG, and PS lipid classes, with total abundance greater in WS and SC samples when compared to SP samples. The PI lipid class showed significance in the *post hoc* comparison between SP and WS as well as SC and WS, with total abundance greatest in WS and total abundance lower in SC and SP samples. Five lipid classes were most prevalent in SC samples compared with WS and SP samples: AC, CER, DG 18:0, DG 18:1, PC, and PE. Three lipid classes were most prevalent in WS samples compared with SC and SP samples: PG, PI, and PS. None of the lipid classes were most prevalent in SP samples when compared to SC and WS samples, indicating the SP fraction contained lower lipid levels than the WS and SC fractions.

### 3.2 Lipid class distribution across sperm mobility phenotypes and timepoints

**FIGURE 2:**
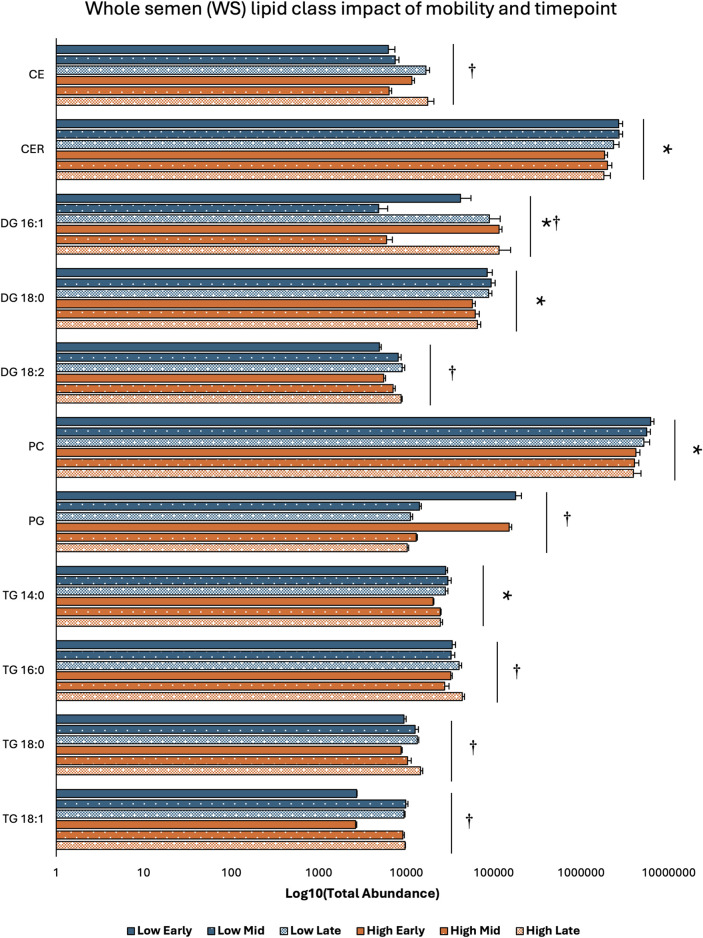
Impacts of sperm mobility phenotype and timepoint on lipid class total abundance in whole semen (WS). Lipid class total abundance differences among sperm mobility phenotypes and semen production timepoints in whole semen (WS) samples. The following lipid classes were analyzed during the screening phase: acylcarnitine (AC), cholesteryl ester (CE), ceramides (CER), diacylglycerol containing a 16:0 fatty acyl chain (DG 16:0), 16:1 (DG 16:1), 18:0 (DG 18:0), 18:1 (DG 18:1), and 18:2 (DG 18:2), phosphatidylcholine (PC), phosphatidylethanolamine (PE), phosphatidylglycerol (PG), phosphatidylinositol (PI), phosphatidylserine (PS), and triacylglycerol containing a 14:0 fatty acyl chain (TG 14:0), 16:0 (TG 16:0), 16:1 (TG 16:1), 18:0 (TG 18:0), 18:1 (TG 18:1), 18:2 (TG 18:2), and 20:0 (TG 20:0). Asterisk symbols denote significant differences due to sperm mobility phenotype while dagger symbols denote significant differences due to timepoint.

**FIGURE 3:**
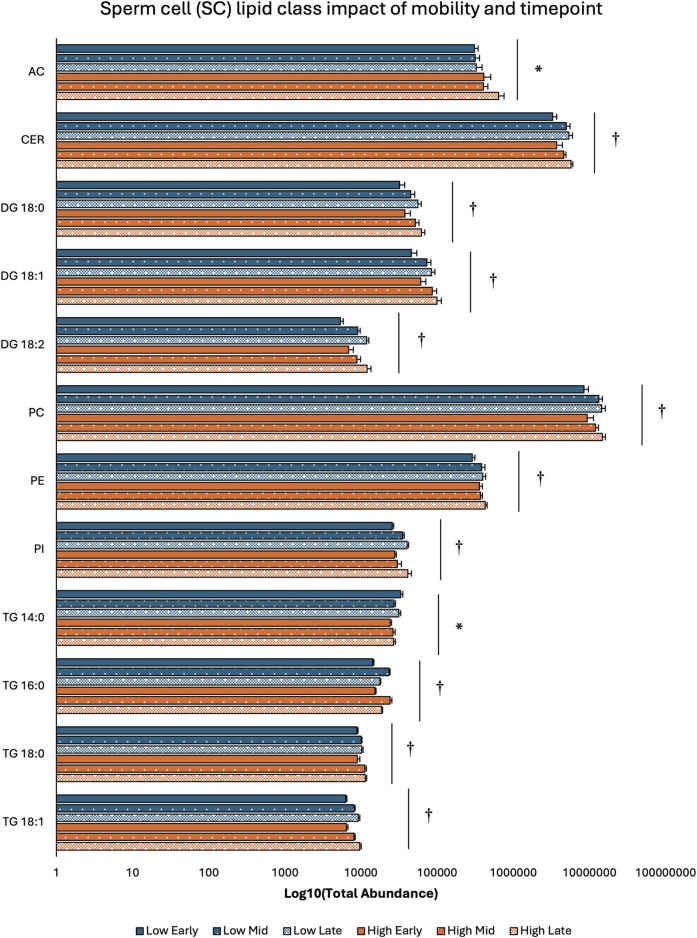
Impacts of sperm mobility phenotype and timepoint on lipid class total abundance in sperm cells (SC). Lipid class total abundance differences among sperm mobility phenotypes and semen production timepoints in sperm cell (SC) samples. The following lipid classes were analyzed during the screening phase: acylcarnitine (AC), cholesteryl ester (CE), ceramides (CER), diacylglycerol containing a 16:0 fatty acyl chain (DG 16:0), 16:1 (DG 16:1), 18:0 (DG 18:0), 18:1 (DG 18:1), and 18:2 (DG 18:2), phosphatidylcholine (PC), phosphatidylethanolamine (PE), phosphatidylglycerol (PG), phosphatidylinositol (PI), phosphatidylserine (PS), and triacylglycerol containing a 14:0 fatty acyl chain (TG 14:0), 16:0 (TG 16:0), 16:1 (TG 16:1), 18:0 (TG 18:0), 18:1 (TG 18:1), 18:2 (TG 18:2), and 20:0 (TG 20:0). Asterisk symbols denote significant differences due to sperm mobility phenotype while dagger symbols denote significant differences due to timepoint.

**FIGURE 4:**
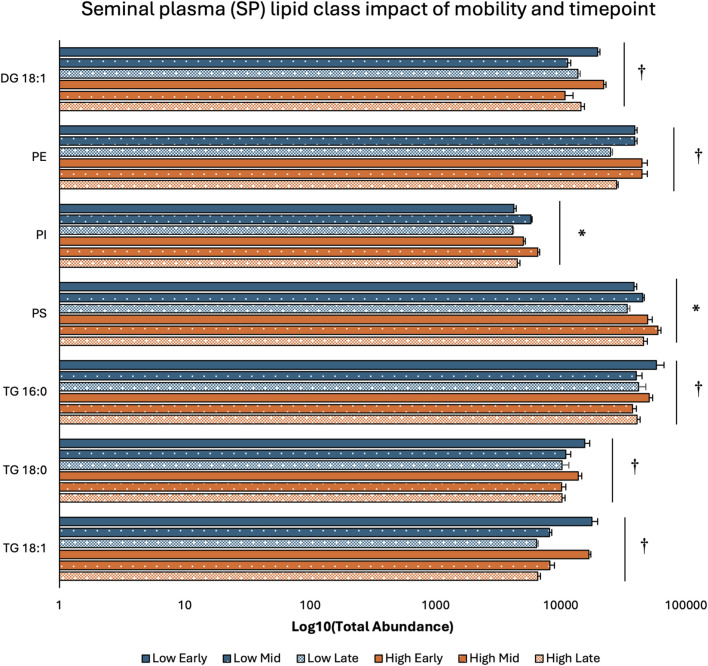
Impacts of sperm mobility phenotype and timepoint on lipid class total abundance in seminal plasma (SP). Lipid class total abundance differences among sperm mobility phenotypes and semen production timepoints in seminal plasma (SP) samples. The following lipid classes were analyzed during the screening phase: acylcarnitine (AC), cholesteryl ester (CE), ceramides (CER), diacylglycerol containing a 16:0 fatty acyl chain (DG 16:0), 16:1 (DG 16:1), 18:0 (DG 18:0), 18:1 (DG 18:1), and 18:2 (DG 18:2), phosphatidylcholine (PC), phosphatidylethanolamine (PE), phosphatidylglycerol (PG), phosphatidylinositol (PI), phosphatidylserine (PS), and triacylglycerol containing a 14:0 fatty acyl chain (TG 14:0), 16:0 (TG 16:0), 16:1 (TG 16:1), 18:0 (TG 18:0), 18:1 (TG 18:1), 18:2 (TG 18:2), and 20:0 (TG 20:0). Asterisk symbols denote significant differences due to sperm mobility phenotype while dagger symbols denote significant differences due to timepoint.

Two-way ANOVA results examining lipid class total abundance differences due to sperm mobility phenotype and timepoint showed total abundance of 7 and 10 lipid classes differed with the main effects of sperm mobility phenotype and timepoint, respectively, in SP, SC, and WS samples, with significant interactions identified in 2 lipid classes from the WS samples. Within WS samples, total abundance of CER, DG 18:0, PC, and TG 14:0 lipid classes were higher in low sperm mobility samples while total abundance of DG 16:1 lipid class was higher in high sperm mobility samples. Additionally, in WS samples, total abundance of CE, DG 16:1, PG, TG 16:0, and TG 18:1 lipid classes peaked at the late timepoint, while total abundance of TG 18:0 and DG 18:2 lipid classes peaked at the early timepoint (Figure 2). Within SC samples, total abundance of AC and TG 14:0 lipid classes were higher in high and low sperm mobility samples, respectively. In terms of timepoint, SC total abundance of CER, DG 18:0, DG 18:1, DG 18:2, PC, PE, PI, TG 18:0, and TG 18:1 lipid classes increased with time, while SC total abundance of the TG 16:0 lipid class peaked at the mid timepoint (Figure 3). In SP samples, total abundance of PI and PS lipid classes was higher in high sperm mobility samples compared to low sperm mobility samples. SP total abundance of DG 18:1, PE, TG 16:0, TG 18:0 and TG 18:1 lipid classes peaked at the early timepoint, PI and PS lipid classes peaked at the mid timepoint, and the AC lipid class while peaked at the late timepoint (Figure 4). Total abundance levels of lipid classes not mentioned above were not different among sperm mobility phenotypes or timepoints.

### 3.3 Differentially abundant lipid species due to mobility

T-test comparison of screening phase lipid species in sperm cell, seminal plasma, and whole semen samples at each timepoint revealed a total of 198 individual lipid species that differed between sperm mobility phenotypes, with overlap of significant lipid species across the semen fractions and timepoints. The largest number of lipid species differences between low mobility and high mobility samples were observed in whole semen samples, with 77, 34, and 1 lipid species determined at the early, mid, and late sampling timepoints, respectively (Supplementary File S1). Sperm cell samples showed minimal lipid species differences between low and high mobility samples at the early and mid-sampling timepoints, with only 11 lipid species at each timepoint, but 77 lipid species differences at the late timepoint (Supplementary File S1). The least number of lipid species differences between low mobility and high mobility samples were observed in seminal plasma samples with 8, 12, and 11 lipid species determined at the early, mid, and late sampling timepoints, respectively (Supplementary File S1).

**FIGURE 5:**
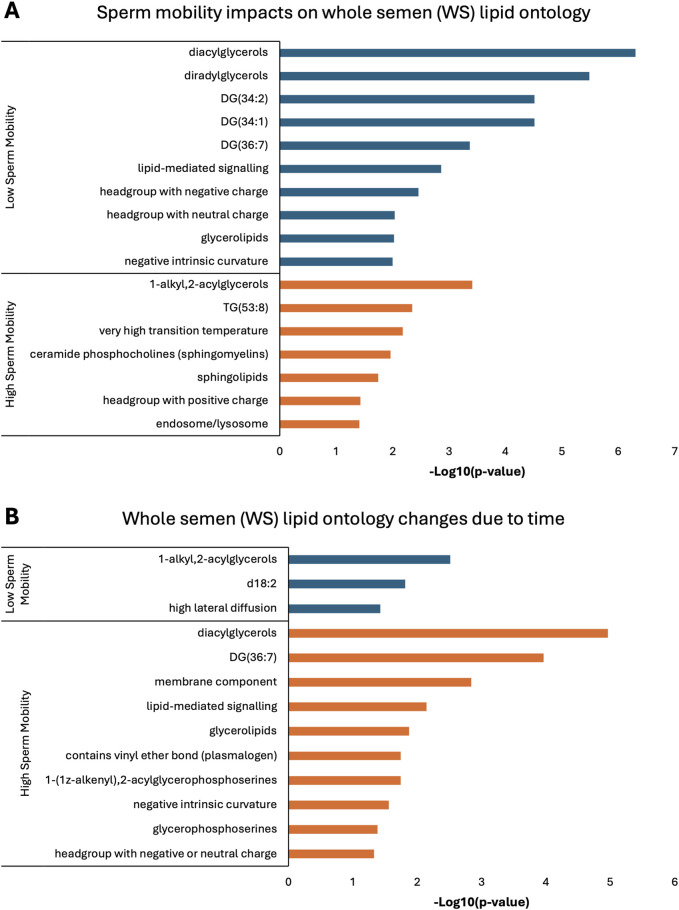
Biological functions of lipid species in whole semen (WS). Enriched lipid ontology terms generated from lipid ontology enrichment analysis (LION) of lipid species significant due to **(A)** sperm mobility phenotype and **(B)** timepoint in the semen production cycle.

**FIGURE 6:**
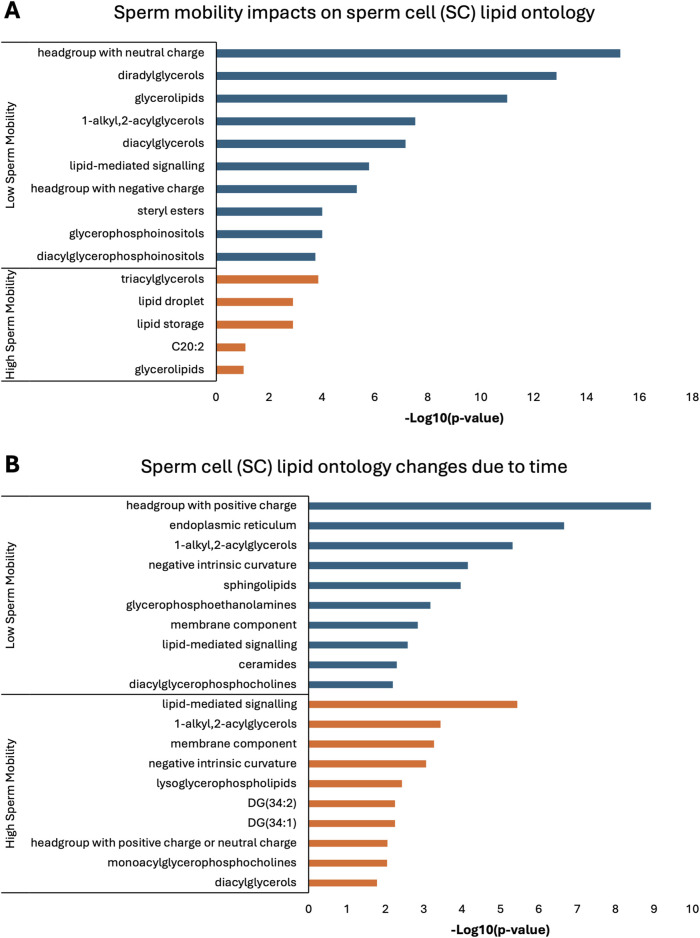
Biological functions of lipid species in sperm cells (SC). Enriched lipid ontology terms generated from lipid ontology enrichment analysis (LION) of lipid species significant due to **(A)** sperm mobility phenotype and **(B)** timepoint in the semen production cycle.

**FIGURE 7:**
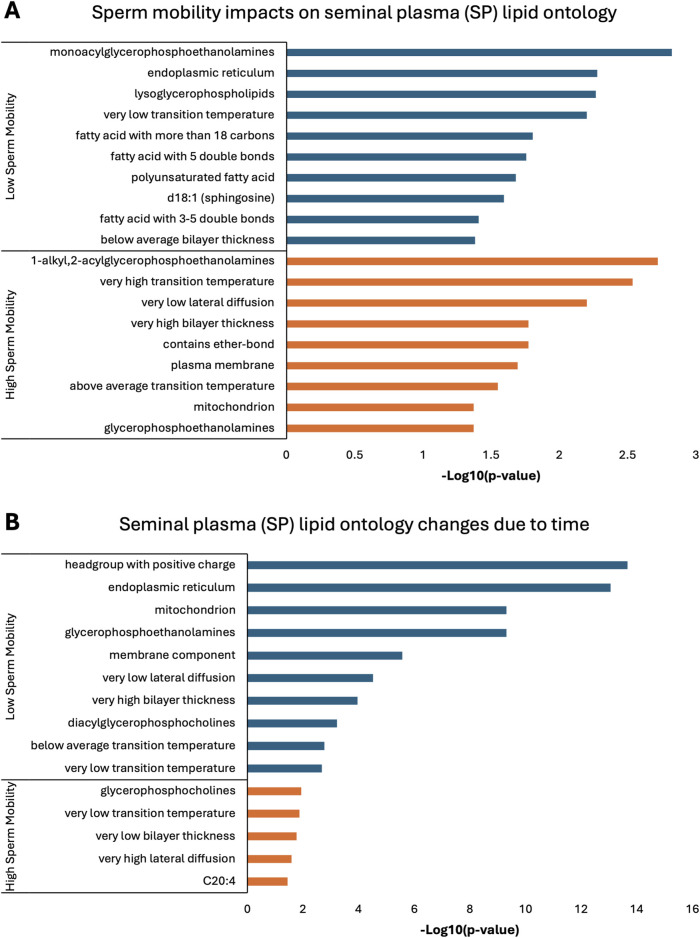
Biological functions of lipid species in seminal plasma (SP). Enriched lipid ontology terms generated from lipid ontology enrichment analysis (LION) of lipid species significant due to **(A)** sperm mobility phenotype and **(B)** timepoint in the semen production cycle.

Lipid ontology enrichment analysis (LION) revealed lipid species with increased abundance in low sperm mobility WS and SC samples showed enrichment for diacylglycerols, lipid-mediated signaling, and negative or neutral charge headgroups. On the other hand, lipid species with increased abundance in high sperm mobility WS and SC samples showed enrichment for triacylglycerols, sphingolipids, and positive charge headgroups (Figures 5A, 6A). LION analysis of lipid species that differed in SP samples from low and high sperm mobility roosters revealed unique lipid ontology terms when compared to those obtained through analysis of SC and WS samples. LION analysis showed abundant lipid species in low sperm mobility SP samples were enriched for lysoglycerophospholipids, very low transition temperature, polyunsaturated fatty acids, and below average bilayer thickness. Conversely, abundant lipid species in high sperm mobility SP samples were enriched for very high transition temperature, very low lateral diffusion, very high bilayer thickness, and positive charge headgroups. SP lipid species differentiating between sperm mobility phenotypes were enriched for derivatives of glycerophosphoethanolamines, with low and high sperm mobility samples showing enrichment for monoacylglycerophosphoethanolamines and 1-alkyl,2-acylglycerophosphoethanolamines, respectively (Figure 7A).

### 3.4 Differentially abundant lipid species due to timepoint

A total of 249 individual lipid species differed among timepoints, with 146 lipid species uniquely identified in low sperm mobility rooster samples, 51 lipid species uniquely identified in high sperm mobility rooster samples and 52 lipid species overlapping between the two sperm mobility phenotypes. Samples originating from low and high sperm mobility roosters showed unique changes in lipid species over time, with a degree of overlap observed in SC samples and minimal overlap in WS and SP samples. For high sperm mobility roosters, 20, 81, and 3 lipid species differed among timepoints in WS, SC, and SP samples, respectively (Supplementary File S2). On the other hand, in low sperm mobility roosters, 18, 144, and 75 lipid species differed among timepoints in WS, SC, and SP samples, respectively (Supplementary File S2). Within WS samples, no overlap was observed between low and high sperm mobility samples. Additionally, *post hoc* comparison showed WS lipid species changes were primarily observed in early to mid-timepoint comparisons in high sperm mobility samples while lipid species changes were primarily observed in early to late-timepoint comparisons in low sperm mobility samples. In SC samples, 41 lipid species overlapped between low and high sperm mobility samples timepoint analyses. Post-hoc comparison of SC lipid species changes over time revealed observed changes were primarily localized to the early to late timepoint comparisons in both low and high sperm mobility samples. Only 1 lipid overlapped between low and high sperm mobility samples timepoint analyses of SP samples. In both low and high sperm mobility SP samples, *post hoc* comparison revealed observed changes were primarily localized to the early to mid-timepoint comparisons.

In low sperm mobility WS samples, high lateral diffusion and 1-alkyl,2-acylglycerols were among the enriched LION terms associated with time, while in high sperm mobility WS samples, diacylglycerols, membrane components, lipid mediated signaling, and plasmalogens were among the enriched LION terms associated with time (Figure 5B). In SC samples, low and high sperm mobility rooster lipid species associated with time exhibited enrichment for the following terms: DG (34:1) and (34:2), negative intrinsic curvature, membrane components, 1-alkyl,2-acylglycerols, lipid-mediated signaling, and headgroup with positive charge. Unique terms enriched in low sperm mobility SC samples included diacylglycerophosphocholines, diacylglycerophosphoethanolamines, sphingolipids such as ceramides, and endoplasmic reticulum. On the other hand, unique terms enriched in high sperm mobility SC samples included monoacylglycerophosphocholines, lysoglycerophospholipids, and headgroup with neutral charge (Figure 6B). Lastly, in SP samples, overlap was observed in very low transition temperature and glycerophosphocholines LION terms between lipid species associated with time in low and high sperm mobility samples. Conversely, LION terms involving lateral diffusion and bilayer thickness contrasted in low and high sperm mobility SP samples. In addition, low sperm mobility SP samples showed enrichment of membrane components, glycerophosphoethanolamines such as diacylglycerophosphocholines, mitochondrion, endoplasmic reticulum, and headgroup with positive charge LION terms, which were not observed in high sperm mobility SP samples (Figure 7B).

### 3.5 Biomarker analysis

**FIGURE 8:**
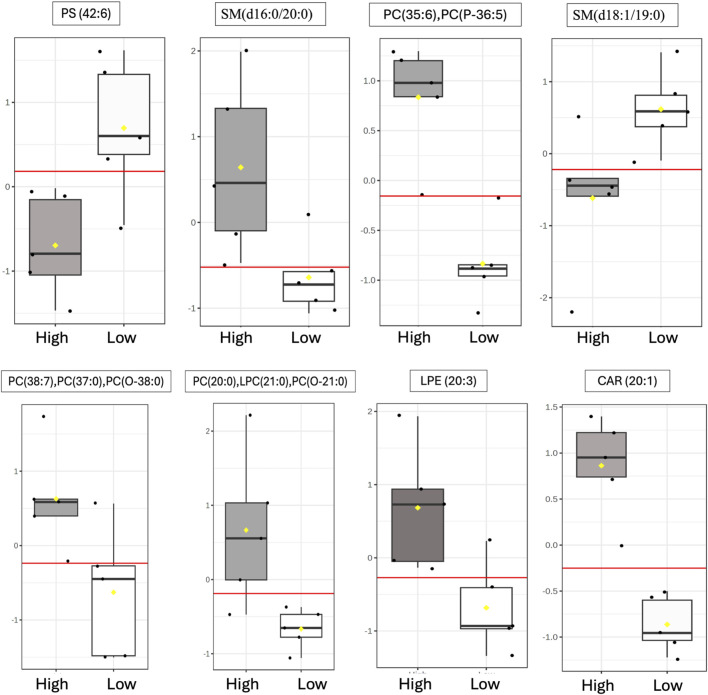
Identification of putative sperm mobility biomarkers in whole semen. Distribution during the early timepoint of differentially abundant lipids within high vs low mobility males following ROC curve analysis that maintained excellent biomarker status through the Mid timepoints. The following AUC scale was used to evaluate lipids as potential biomarkers: greater than or equal to 0.9 scored as excellent; greater than or equal to 0.8 but less than 0.9 scored as good; greater than or equal to 0.7 but less than 0.8 scored as fair; greater than or equal to 0.6 but less than 0.7 scored as poor; and less than 0.6 scored as fail. The yellow notches within the distribution plots represent the 95% confidence interval around the median for each group. If the notches do not line up, the medians are likely different. The red line represents the median. The *y*-axis for box plots represents the relative ion intensity of lipids within each sample.

ROC curve analysis was performed to determine the potential of lipid species signatures in whole semen as biomarkers of sperm mobility. There were 8 lipid species identified as excellent biomarkers (AUC >0.9) during the early timepoint that could be tested through the mid timepoint, with these lipid species maintaining an AUC greater than 0.8 in the mid timepoint as well (Table 1). Lysophosphatidylethanolamine (20:3), eicoseneoylcarnitine, and phosphatidylserine (42:6) maintained their status as excellent biomarkers through the mid timepoint. Further, all lipids during the late timepoint either became poor or failed biomarkers. Two of the eight lipids, sphingomyelin (d18:1/19:0) and phosphatidylserine (42:6), were higher in abundance and indicative of low mobility during the early period. The remainder of the potential biomarkers of sperm mobility were predictive of high mobility (Figure 8).

**TABLE 1:** Relative difference and biomarker analysis between high and low mobility phenotype in lipid abundance from whole semen collected at different timepoints.

Method	Tentative ID attribution/MRM ID	Lipid class	Common name	High--low FC^a^ (log_2_)	*p*-value	AUC^b^	Timepoint^c^
M1	LPE (20:3)	Glycerophosphoethanolamine	Lysophosphatidylethanolamine (20:3)	0.68	0.02	0.92	Early
				0.42	0.03	0.96	Mid
	PC(20:0),LPC(21:0),PC(O-21:0)	Glycerophosphocholine	PC(20:0),LPC(21:0),PC(O-21:0)	0.44	0.02	0.92	Early
				0.36	0.04	0.84	Mid
	PC(35:6),PC(P-36:5)	Glycerophosphocholine	PC(35:6),PC(P-36:5)	0.60	<0.01	1.00	Early
				0.51	0.03	0.88	Mid
	PC(38:7),PC(37:0),PC(O-38:0)	Glycerophosphocholine	PC(38:7),PC(37:0),PC(O-38:0)	0.25	0.04	0.92	Early
				0.18	0.11	0.84	Mid
	SM(d16:0/20:0)	Sphingomyelin	Sphingomyelin (d16:0/20:0)	0.50	0.03	0.92	Early
				0.50	0.06	0.84	Mid
	SM(d18:1/19:0)	Sphingomyelin	Sphingomyelin (d18:1/19:0)	−0.15	0.04	0.92	Early
				0.22	0.05	0.84	Mid
M3	CAR(20:1)	Acylcarnitine	(11Z)-eicoseneoylcarnitine	1.99	<0.01	1.00	Early
				0.27	0.01	0.92	Mid
	PS(42:6)	Glycerophosphoserine	Phosphatidylserine (42:6)	−0.34	0.02	0.92	Early
				0.22	0.01	1.00	Mid

^a^ Fold Change.

^b^ Area under the curve (AUC) from ROC, curve analysis to evaluate potential mobility biomarkers within whole semen.

^c^ Males were collected at three different timepoints: Early, Mid, and Late. all lipids within this table lost their biomarker potential by the late timepoint.

## 4 Discussion

The overall goal of this study was to examine the lipidome of poultry semen fractions using MRM profiling, with special focus on lipid shifts due to sperm mobility phenotype and timing in the semen production cycle. This study is the first to characterize the lipidome of WS, SC, and SP in broiler breeder roosters. Further, this study identified and biologically analyzed 198 lipid species associated with sperm mobility phenotype and 249 lipid species associated with timing in the semen production cycle. Lastly, this study determined 8 potential biomarkers that scored as excellent biomarkers of the sperm mobility phenotype in early semen production, with these biomarkers remaining excellent or good throughout mid-semen production as well. Results from this study are imperative for determining how semen lipid composition intersects with sperm mobility as well as how this lipid composition changes throughout the sperm production cycle, ultimately leading to reduced sperm mobility with rooster age. Biomarkers identified will require further evaluation but are an important milestone moving the broiler breeder industry closer to mid-high throughput options for fertility potential screening in roosters.

Unique lipid class total abundances were found in the SP and SC, with WS integrating aspects of both fractions. The most prevalent lipid classes identified in poultry semen were CER, PC, and PE, with increased abundance in SC and WS fractions compared to the SP fraction. Ceramides are known to increase membrane rigidity and participate in membrane diffusion of small solutes, such as proteins (Alonso and Goñi, 2018; Farrani and Maggio, 2017; Alonso and Goñi, 2018). Additionally, ceramides contribute to lipid traffic across membranes through their participation in lipid rafts (Pinto et al., 2011). Phosphatidylcholine and phosphatidylethanolamine are the most important components of the sperm plasma membrane, with abundance levels and ratios serving as indicators of sperm fertilization potential (Shan et al., 2020). The high total abundance of ceramide, phosphatidylcholine, and phosphatidylethanolamine lipid classes in poultry semen, all of which play key roles in the sperm membrane composition and function, is consistent with the high density of sperm cells in poultry when compared to mammalian semen. Further, similar lipid class proportions have been previously reported for poultry semen, confirming that MRM profiling results are consistent with less robust technologies that are unable to resolve down to the lipid species level.

Low sperm mobility roosters exhibited increased total abundance of CER, PC, DG 18:0, and TG 14:0 lipids, while high sperm mobility roosters exhibited increased total abundance of DG 16:1, AC, PI, and PS lipids. Several studies in mammals have identified key CER and PC lipids in semen associated with reduced male performance, while increased TG 14:0 lipids were associated with morphological defects in swine semen (Chen et al., 2021; Shan et al., 2020; Mills et al., 2024). Additionally, PC and AC lipid levels were negatively associated with poultry sperm motility/mobility while PS lipid levels were positively associated (Cerolini et al., 1997; Froman and Kirby, 2005). Interestingly, the identified lipid classes play diverse roles in sperm physiological functions, from sperm cell membrane integrity/fluidity to lipid based signaling to sperm energy sources. This could indicate that sperm mobility phenotypes result from a culmination of several physiological mechanisms. Though PI lipids display low abundance in poultry semen, phosphoinositide signaling has been identified as imperative for sperm motility as well as SST metabolic processes in poultry, indicating potential mechanistic links between increased SP PI levels and increased sperm mobility (Lemoine et al., 2009; Yang et al., 2021). Diglycerides play vast roles in sperm function, including key roles in the acrosome reaction and sperm metabolism. Mammalian research results on diglyceride impacts on sperm fertility potential are mixed, with reports of increased diglyceride levels in high fertility and low fertility males, similar to the results from this study (Shan et al., 2020; Sebastian et al., 1987). In addition to sperm mobility-based differences, each lipid class examined differed among semen production cycle timepoints, indicating that the lipidome of broiler breeder rooster semen is dynamic. In WS and SC samples, a majority of the lipid classes were most abundant at the late timepoint, while in SP samples, 5 lipid class abundances peaked at the early timepoint. Early SP lipid changes could serve to drive later changes in SC/WS, ultimately influencing sperm mobility. The lipid class changes due to age indicated in this study do not completely align with previous broiler breeder reports from 2 decades ago (Kelso et al., 1996). This could be due to line-based differences and/or that intensive genetic selection has shifted the lipid composition of broiler breeder semen.

LION analysis of lipid species that differed between low and high sperm mobility phenotypes revealed stark contrasts in transition temperature, lateral diffusion, and bilayer thickness. Transition temperature, bilayer thickness, and lateral diffusion are tightly linked to lipid bilayer membrane fluidity and integrity, which regulate nutrient availability, trans-membrane transport proteins activity, and intracellular signaling. Semen transition temperature has been showed to be reduced in species less resistant to temperature changes (Drobnis et al., 1993). Increased bilayer thickness increased trans-membrane transport proteins activity, for ions such as calcium which impacts sperm mobility, up to a peak prior to decreasing activity once the bilayer threshold has been obtained (Johannsson et al., 1981). High sperm mobility samples exhibited increased transition temperature and bilayer thickness, potentially increasing temperature stability and calcium trans-membrane transport in semen obtained from these roosters. Additionally, low sperm mobility samples exhibited enrichment of lateral diffusion, a process increasing membrane fluidity that is required during later stages of sperm-ovum interaction (Wolfe et al., 1998). This premature shift may leave low mobility semen incapable of membrane fluidity changes at later stages of fertilization. Overall, low sperm mobility semen fractions exhibited changes in a larger number of lipid species due to timing in the semen production cycle than high sperm mobility semen fractions, particularly in SC and SP samples. In SC samples, LION terms related to lipid signaling and membrane components appear enriched due to timepoint in both low and high sperm mobility phenotypes, implying similar time-based shifts unrelated to sperm mobility. In SP samples, LION terms related to bilayer thickness and lateral diffusion appear again, with high sperm mobility samples shifting with time toward lower bilayer thickness and higher lateral diffusion as was previously observed in low sperm mobility samples. Through lipid species changes during the semen production cycle, high and low sperm mobility samples appear to converge and become more similar at later timepoints. This is also supported by the apparent reduction in the number of lipid species differing between the two sperm mobility phenotypes, with the largest differences observed during the early timepoint and nearly no differences observed by the late timepoint. A trend toward convergence in also observed in broiler breeder sperm mobility scores over time, with low sperm mobility roosters improving sperm mobility scores with time while high sperm mobility roosters remain constant (Bowling et al., 2003).

ROC curve analysis identified three excellent biomarkers associated with mobility that can be tested beginning at 30 weeks (early) through 42 weeks of age (mid). LPE (20:3) and CAR (20:1) were associated with high mobility and PS (42:6) with low mobility at 30 weeks of age. CAR (20:1), also known as eicoseneoylcarnitine, is a structural derivative of carnitine that acts as a metabolite for cellular energy and metabolism (Degtyarenko et al., 2007). Carnitines are naturally occurring antioxidants and have been documented to be important for sperm maturation, with L-carnitine levels high within the epididymis, as well as sperm metabolism, with sperm cells utilizing fatty acid oxidation for energy supply (Soufir et al., 1981; Jeulin et al., 1987). In young broiler breeder roosters aged 24–34 weeks, increasing levels of supplemented L-carnitine resulted in an increase in sperm concentration, live sperm, and sperm quality factor—another fertility index for male poultry (Mohammadi et al., 2021). This data agrees with other work done in aging roosters where increased L-carnitine supplementation improved overall semen quality and sperm quality factor (Elokil et al., 2019). CAR (20:1) may serve to improve energy availability for sperm metabolism and may support sperm maturation at the level of the epididymis. In birds, successful fertilization can only occur when the sperm cells enter or be stored within SST (Alexander et al., 1993). Modifications of carbohydrates and phospholipids within the sperm plasma membrane decreases the capacity to survive selection and storage within SSTs, thus reducing fertility (Froman and Thurston, 1984; Donoghue et al., 1995). In the rooster sperm cell head and body membranes, PC and PE lipids are prevalent, with PS being the smallest lipid class (Bongalhardo et al., 2002). LPE (20:3) and PS (42:6) are both sperm plasma membrane lipids and their modifications or ratios within individual rooster could lead to downstream sperm mobility phenotype differences. With PS established as a less prevalent lipid class in poultry semen, the higher abundance of PS (42:6) in low mobility males may result in decreased mobility.

Historically, low sperm mobility leading to decreased fertility potential has been a detriment to the broiler breeder industry. From the standpoint of the producer, fertility prediction as early as possible is advantageous as this reduces economic losses incurred by male fertility challenges. Thus, having an early-detectable biomarker or biomarker panel indicative of fertility potential would be highly valuable to the broiler breeder industry. The ability to perform early semen lipidome biomarker screening to detect sperm mobility phenotypes would assist in mitigating the financial disparities stemming from low sperm mobility roosters. Results from this study determined differences in lipid class abundance and individual lipid species between low and high sperm mobility rooster semen fractions. Eight of the lipid species identified in WS also scored highly as putative biomarkers of the sperm mobility phenotype. However, this study also indicated age-based associations on the lipid composition of poultry semen fractions, which is important to consider when determining when biomarker screening can be performed. Additional research is needed to validate putative biomarkers of the sperm mobility phenotype and to determine acceptable screening timelines that allow reliable results, ultimately allowing for industry-based selection strategies to improve broiler breeder rooster fertility potential.

## Data Availability

The original contributions presented in the study are included in the article/Supplementary Material, further inquiries can be directed to the corresponding author.
